# Myosin Light Chain Kinase: A Potential Target for Treatment of Inflammatory Diseases

**DOI:** 10.3389/fphar.2017.00292

**Published:** 2017-05-23

**Authors:** Yongjian Xiong, Chenou Wang, Liqiang Shi, Liang Wang, Zijuan Zhou, Dapeng Chen, Jingyu Wang, Huishu Guo

**Affiliations:** ^1^Central Laboratory, The First Affiliated Hospital, Dalian Medical UniversityDalian, China; ^2^Laboratory Animal Center, Dalian Medical UniversityDalian, China

**Keywords:** myosin light chain kinase, inflammatory bowel diseases, cancer, tight junctions, endothelium

## Abstract

Myosin light chain kinase (MLCK) induces contraction of the perijunctional apical actomyosin ring in response to phosphorylation of the myosin light chain. Abnormal expression of MLCK has been observed in respiratory diseases, pancreatitis, cardiovascular diseases, cancer, and inflammatory bowel disease. The signaling pathways involved in MLCK activation and triggering of endothelial barrier dysfunction are discussed in this review. The pharmacological effects of regulating MLCK expression by inhibitors such as ML-9, ML-7, microbial products, naturally occurring products, and microRNAs are also discussed. The influence of MLCK in inflammatory diseases starts with endothelial barrier dysfunction. The effectiveness of anti-MLCK treatment may depend on alleviation of that primary pathological mechanism. This review summarizes evidence for the potential benefits of anti-MLCK agents in the treatment of inflammatory disease and the importance of avoiding treatment-related side effects, as MLCK is widely expressed in many different tissues.

In mammals, myosin light chain kinase (MLCK) is encoded by the *mylk1* and *mylk2* genes (Herring et al., 2006). *mylk2* encodes an MLCK isoform that is exclusively expressed in skeletal muscle cells (Herring et al., 2006; Wang L. et al., 2016). Because of the lack of data on *mylk2* gene coding products, we mainly discuss *mylk1* gene products, which include long chain MLCK (220 kDa), short chain MLCK (130 kDa), and the non-catalytic carboxy-terminal (17 kDa) protein, telokin (Chen et al., 2013; Chen C. et al., 2014; An et al., 2015). *mylk1* gene coding products are expressed in diverse cell types and tissues including muscle, platelets, and secretory and brain cells (Jin et al., 2002). Numerous cell activities, such as contraction, adhesion, cell migration, and epithelial barrier formation occur in a myosin regulatory light chain (MLC) phosphorylation dependent or independent manner (Chen et al., 2013; Chen C. et al., 2014; Kim and Helfman, 2016). Abnormal expression of MLCK has been observed in many inflammatory diseases including pancreatitis (Shi et al., 2014), respiratory diseases (Zhou et al., 2015), cardiovascular diseases (Cheng et al., 2015), cancer (Zhou et al., 2014), and inflammatory bowel disease (IBD) (Yi et al., 2014). The involvement of MLCK and the MLCK signaling pathway that underlie representative inflammatory diseases is discussed. Some diseases in which MLCK is involved are listed in **Table 1**.

**Table 1:** Role of myosin light chain kinase (MLCK) in selected diseases.

Diseases	MLCK changes	MLCK isoform	Representative References
Atherosclerosis	Increased expression	nmMLCK	Zhu et al., 2013
Hypertension	Increased activity	smMLCK	Cho et al., 2011
Heart injury/Heart failure	Increased activity	Cardiac MLCK	Lin et al., 2012; Chang et al., 2013
Glaucoma	Increased activity	smMLCK	Prayitnaningsih et al., 2016
Asthma	Increased expression/Gene variant	nmMLCK	Zhou et al., 2015
Lung inflammation/Lung injury	Increased expression /Gene variant	nmMLCK	Mirzapoiazova et al., 2009; Wu et al., 2011
Brain injury /Kidney injury	Increased expression	nmMLCK	Xu et al., 2015; Droylefaix et al., 2013
Intestinal inflammation /IBD/Barrier dysfunction	Increased expression	nmMLCK	Du et al., 2016; Jin and Blikslager, 2016; Xiong et al., 2016
Intestinal motility disorder	Increased/Decreased expression	smMLCK	Chen et al., 2015
Pancreatitis	Increased	nmMLCK	Shi et al., 2014
Prostate Cancer	–	nmMLCK	Spans et al., 2014
Breast cancer	–	nmMLCK	Kim and Helfman, 2016
Pancreatic cancer	–	nmMLCK	Kaneko et al., 2002
Non-small cell lung cancer	Increased	nmMLCK	Minamiya et al., 2005
Cervical cancer	–	nmMLCK	Shen et al., 2002
Gastric cancer	–	nmMLCK	Chen et al., 2016

*nmMLCK, non-muscle MLCK; smMLCK, smooth muscle MLCK*.

## MLCK in Respiratory Diseases, Atherosclerosis, and Pancreatitis

In inflammatory lung disorders, damage to lung endothelial cell barrier integrity alters vascular permeability, and alveolar flooding often results (Mao et al., 2015). Abnormal expression of MLCK occurs in lung injury, and the MLCK inhibitor ML-7 or deletion of the *MLCK* gene can attenuate lung injury (Wang T. et al., 2016). MLCK has similar activity in asthmatic and in lung inflammation, and variation of the *MYLK* gene is strongly associated with acute lung injury and asthma susceptibility (Wang et al., 2014, 2015; Wang T. et al., 2016).

MLCK-induced endothelial barrier dysfunction is also involved in pancreatitis and atherosclerosis (Cheng et al., 2015; Wang et al., 2014; Wang T. et al., 2016). Severe acute pancreatitis is associated with high morbidity and mortality. Its pathogenesis is not completely understood (Zerem, 2014), but MLCK expression is significantly increased in rat models of acute pancreatitis (Shi et al., 2014), and elevation of tumor necrosis factor (TNF)-α in severe acute pancreatitis has been shown to mediate MLCK-dependent regulation of the cytoskeleton, leading to destruction of the endothelial barrier function (Shi et al., 2014; Yu et al., 2016). The initiation and development of atherosclerosis often leads to progressive vascular injury, which is accompanied by endothelial dysfunction (Phinikaridou et al., 2015). The involvement of MLCK in the natural history of atherosclerosis has been confirmed by alleviation of vascular injury and atherosclerosis by ML-7, an MLCK inhibitor (Cheng et al., 2015).

## MLCK in Cancer Development

Abnormal expression of MLCK has been observed in pancreatic, lung, and prostate cancer cell lines (Tohtong et al., 2003; Nagaraj et al., 2010; Chen et al., 2011). Rapid, dynamic changes of the cytoskeleton are needed for invasion and metastasis of cancer cells. MLCK-dependent phosphorylation of cytoskeletal myosin II increases the metastatic potential of tumor cells, and MLCK-dependent cytoskeleton rearrangement modulates vascular endothelial barrier functions associated with angiogenesis, which is a critical step in cancer development (Dudek and Garcia, 2001). On the other hand, the metastatic potential of breast cancer cells is increased by the loss of MLCK (Kim and Helfman, 2016). Changes in cell migration and adhesion are also characteristic early steps in inflammation but there are few reports of MLCK regulation of inflammatory cell migration.

## MCLK in IBD

Inflammatory bowel disease, including ulcerative colitis and Crohn’s disease, is characterized by chronic gastrointestinal inflammation, and is associated with significant patient impairment and high treatment costs (Rai et al., 2015). Although the pathogenesis of IBD remains obscure, there is evidence that intestinal barrier dysfunction is the primary driver (Hindryckx and Laukens, 2012; Pastorelli et al., 2015). Tight junction dysfunction leads to damage of the intestinal barrier, which permits passage of diverse pathogens (Jin and Blikslager, 2016). Tight junctions consist of transmembrane proteins such as occludins and claudins and peripheral membrane proteins, i.e., zonula occludens proteins (Van Itallie and Anderson, 2014). Tight junctions are located in the apicolateral region of endothelial cells and are bound to a perijunctional actomyosin ring. MLCK-induced phosphorylation of perijunctional actomyosin mediates tight junction loss, which can trigger the initiation and development of IBD. The expression and activity of MLCK is increased in human IBD and is associated with histological evidence of disease activity (Blair et al., 2006). Abnormal elevation of MLCK has also been observed in experimental colitis induced by gavage administration of dextran sulfate sodium or intracolonic administration of trinitrobenzenesulfonic acid (Su et al., 2013; Xiong et al., 2016).

### MLCK Activation in IBD

TNF-α is a proinflammatory cytokine that causes intestinal tight junction barrier dysfunction, which is central to IBD pathogenesis (Saleh et al., 2016). In IBD, TNF receptor 2 (R2)-mediated signaling contributes to increased epithelial MLCK expression (Su et al., 2013; Suzuki et al., 2014). In a recent report by Al-Sadi et al. (2013), tight junction permeability of Caco-2 cell monolayers, in an *in vitro* model of intestinal epithelium, was increased by TNF-α activation of the ERK1/2 signaling pathway. Activation of the ERK1/2 pathway induced phosphorylation of ETS domain-containing transcription factor Elk-1. Activated Elk-1 then moved into the nucleus and bound to the MLCK promoter, finally resulting in epithelial MLCK expression. LIGHT (lymphotoxin-like inducible protein that competes with glycoprotein D for herpes virus entry on T cells) is a TNF core family member that is involved in the pathogenesis of human IBD (Krause et al., 2014), and in cultured epithelia, MLCK inhibition alleviated LIGHT-induced barrier loss, which suggested that LIGHT-induced epithelial barrier loss may depend on MLCK activation (Schwarz et al., 2007).

Increases in tight junction permeability through IL-1β–mediated increases in MLCK expression has been demonstrated in inflammatory diseases (Beard et al., 2014). In mesenchymal stem cell migration, IL-1β was shown to cause an increase in epithelial MLCK expression through activation of the PKCd/NF-κB pathway; it also stimulated MLCK activity via the PKCa/MEK/ERK signaling pathway (Lin et al., 2014).

IFN-γ has also been associated with activation of MLCK by promoting adhesion and internalization of commensal bacteria by epithelial MLCK-activated brush border fanning (Wu et al., 2014). However, as with LIGHT-mediated regulation of MLCK, further study of INF-γ-mediated regulation of MLCK is needed to determine if it is direct. Signaling pathways associated with regulation of MLCK are shown in **Supplementary Figure S1**.

### MLCK-Associated Signaling Pathways That Can Trigger IBD

In IBD, MLCK-induced epithelial barrier dysfunction is triggered by two signaling pathways. Firstly, in the gut, the epithelium forms a barrier against pathogens in the lumen. Abnormal expression of MLCK in inflammatory gastrointestinal diseases leads to phosphorylation of myosin II regulatory light chain (MLC), contraction of the actomyosin ring and increased intestinal permeability (Yi et al., 2015). Thus, MLCK-dependent MLC phosphorylation is an essential mechanism underlying MLCK-induced epithelial barrier dysfunction. A second mechanism involves MLCK-stimulated upregulation of claudin-2 and occludin endocytosis (Su et al., 2013; Jin and Blikslager, 2016). Increased expression of claudin-2 has been associated with intestinal epithelial barrier dysfunction (Hu et al., 2015; Krishnan et al., 2015), as well as decreased absorption, leak flux diarrhea, and inflammatory responses (Hu et al., 2015). Down-regulation of occludin in IBD decreases gastrointestinal permeability, which may disrupt the integrity of the barrier against a variety of pathogens (Yin et al., 2015).

### Potential Pathological Role of Smooth Muscle MLCK in IBD

Smooth muscle (sm) MLCK is transcribed from the same gene as epithelial MLCK. It is involved in the regulation of sm contraction, and variation of smMLCK content leads to motility disorders (Chen et al., 2015). The motility disorders secondarily cause abnormal growth of intestinal flora, which in turn aggravates the pathogenesis of intestinal inflammation (Chen D. et al., 2014; Welch et al., 2014). Whether there is a direct effect of smMLCK on inflammatory diseases needs further study.

## MLCK Inhibitors with Potential Pharmaceutical Use

Myosin light chain kinase has catalytic, inhibitory, and calmodulin-binding domains (Chang et al., 2016). The activity of the catalytic domain can be disclosed by partial tryptic digestion, and can be blocked by MLCK inhibitors (Luck and Choh, 2011; Chang et al., 2016). MLCK inhibitors act by competitive binding at or near the ATP-binding site on the MLCK molecule (Saitoh et al., 1987; Luck and Choh, 2011). MLCK has been extensively studied in sm, but is widely distributed in animal cells and tissues. Consequently, determining the activities of MLCK in other tissues is critical; MLCK inhibitors are good tools for this. MLCK inhibitors also have pharmacological potential as vasodilators and anti-inflammatory agents. Some MLCK inhibitors, their origins and evidence of pharmacological effect are listed in **Table 2**.

**Table 2:** Myosin light chain kinase inhibitors with potential pharmaceutical use.

Name	Source	Inhibited MLCK isoform	Disease or condition
ML-9	Synthetic	nmMLCK, smMLCK	High blood pressure (Honjo et al., 2002).
ML-7	Synthetic	nmMLCK, smMLCK	Heart ischemia/reperfusion injury (Lin et al., 2012; Zhang et al., 2015), IBD (Cheng et al., 2015), and atherosclerosis (Cheng et al., 2015).
K-252a	Microbial culture	nmMLCK, smMLCK	–
KT592	Microbial culture	nmMLCK, smMLCK	–
Wortmannin	Microbial culture	NmMLCK, smMLCK	–
Quercetin	Natural source	SmMLCK	Gut hyper motility (Zhang et al., 2006)
Genistin	Natural source	smMLCK	Intestinal hyper motility (Xiong et al., 2013)
Wogonin	Natural source	nmMLCK	Diseases associated with the development of both inflammatory and tumor (Huang et al., 2015)
Capsaicin	Natural source	smMLCK, nmMLCK	Intestinal motility disorder (Chen et al., 2015)
Salvianolic acid B	Natural source	NmMLCK	IBD (Xiong et al., 2016)
Lithium	Natural source	smMLCK	Intestinal hyper motility (Tang et al., 2010)

*The diseases or conditions in which MLCK inhibition and/or MLCK inhibitors have shown a therapeutic effect are discussed in “Diseases and condition.” nmMLCK, non-muscle MLCK; smMLCK, smooth muscle MLCK*.

### ML-9 and ML-7

ML-9 [1-(5-chloronaphthalene-1-sulfonyl)-1H-hexahydro-1,4-diazepine] is a classical MLCK inhibitor (IC_50_ = 3.8 μM), which was found to inhibit both Ca^2+^-calmodulin–dependent and -independent smMLCK (Saitoh et al., 1987; Shi et al., 2007). Both ML-9 and its synthetic derivatives are good selective inhibitors of smMLCK (Ito et al., 2004). ML-9 has been shown to reduce intraocular pressure in rabbit eyes (Honjo et al., 2002).

Another MLCK inhibitor, ML-7 [1-(5-iodonaphthalene-1-sulphonyl) 1H-hexahydro 1, 4-diazepine hydrochloride], is a membrane-permeable agent (Shi et al., 2007). Both ML-9 and ML-7 are naphthalene sulfonamide derivatives (Shi et al., 2007). ML-7 inhibition is more than 30-fold more potent than that of ML-9 (IC_50_ = 300 nM) (Shi et al., 2007). However, compared with ML-9, specific MLCK inhibition of smMLCK and other MLCK isoforms may be less potent (Saitoh et al., 1987). Beneficial effects of ML-7 has been shown in many conditions including heart ischemia/reperfusion injury (Lin et al., 2012; Zhang et al., 2015), IBD (Cheng et al., 2015), and atherosclerosis (Cheng et al., 2015).

### Microbial Product Inhibitors of MLCK

K-252a, a microbial alkaloid purified from microbial cultures, is a non-selective inhibitor of MLCK (Nakanishi et al., 1992) as well as other protein kinases including protein kinase C and some cyclic nucleotide-dependent protein kinases (Nakanishi et al., 1992). KT592 is a derivative of K-252a with increased selectivity. Wortmannin, isolated and purified from the fungal strain *Talaromyces wortmannin* KY12420, is another microbial product inhibitor of MLCK (Nakanishi et al., 1992), It has been shown to decrease secretory responses in rat adrenal medullary cells through inhibition of MLCK (Warashina, 2000) and to have antifungal, hemorrhagic, and anti-inflammatory activity that may not be related to inhibition of MLCK (Nakanishi et al., 1992). The potential pharmacological effects of these inhibitors warrant further study.

### Naturally Occurring Potential Inhibitors of MLCK

As shown in **Table 2**, some naturally occurring bioactive constituents may be inhibitors of MLCK. In an *in vitro* system including purified myosin and MLCK, quercetin inhibited myosin phosphorylation. The inhibition can be blocked by the MLCK inhibitor ML-7, indicating that quercetin may be a direct MLCK inhibitor (Zhang et al., 2006). In an animal model of gut motility disorder, capsaicin administration significantly decreased MLCK expression, which also implicates MLCK as a target for inhibition by capsaicin (Chen et al., 2015). The inhibition in response to salvianolic acid B may be indirect; other signaling is involved. Salvianolic acid B decreases MLCK expression by upregulation of microRNA1 (Xiong et al., 2016). Upregulation of microRNA-374a, microRNA-155, miR-520c-3p, and miR-1290 has also been found to reduce MLCK expression in various tissues (Adyshev et al., 2013; Weber et al., 2014). Naturally occurring bioactive compounds that act indirectly through microRNAs are an alternative inhibition pathway. However, disease-specific pharmacological experiments are needed to confirm the effects of potential naturally occurring inhibitors of MLCK.

## Summary

This review summarizes the evidence for a role of MLCK in inflammatory diseases, especially IBD. Abnormal expression of MLCK is involved in diverse pathological events, mainly by causing cytoskeletal changes that disrupt epithelial barrier function. The effect of anti-MLCK agents in specific inflammatory diseases depends on the extent to which endothelial function is involved. Prevention of treatment-related side effects is a key consideration because MLCK is abundantly expressed in many tissues. Consideration of two aspects of selectivity helps to anticipate and prevent side effects of MLCK inhibitors. First is the selective inhibition of MLCK and other protein kinases such as protein kinase C and cyclic nucleotide-dependent protein kinase; the other is selective inhibition of the different MLCK isoforms such as smMLCK and nmMLCK. Potential anti-MLCK pharmaceutical agents offer a novel insight into the treatment of inflammatory diseases that differs from traditional anti-inflammatory therapy.

## Author Contributions

Conceived and designed the review: DC. References check: DC, YX, CW, LW, ZZ, and LS. Drafted the paper and revised it critically for important intellectual content: DC, YX, CW, LW, ZZ, and LS. The manuscript has been approved by all the authors.

## Conflict of Interest Statement

The authors declare that the research was conducted in the absence of any commercial or financial relationships that could be construed as a potential conflict of interest.

## Supplementary Material

FIGURE S1 **Mechanisms underlying MLCK-induced regulation of the endothelial barrier function are shown**. Solid arrows indicate direct interaction and dotted arrows indicate indirect interactions.
