# Supplementary figures and images for: Myosin Light Chain Kinase: A Potential Target for Treatment of Inflammatory Diseases
Source: Front Pharmacol. 2017 May 23;8:292. doi: 10.3389/fphar.2017.00292 (PMC5440522; doi:10.3389/fphar.2017.00292)

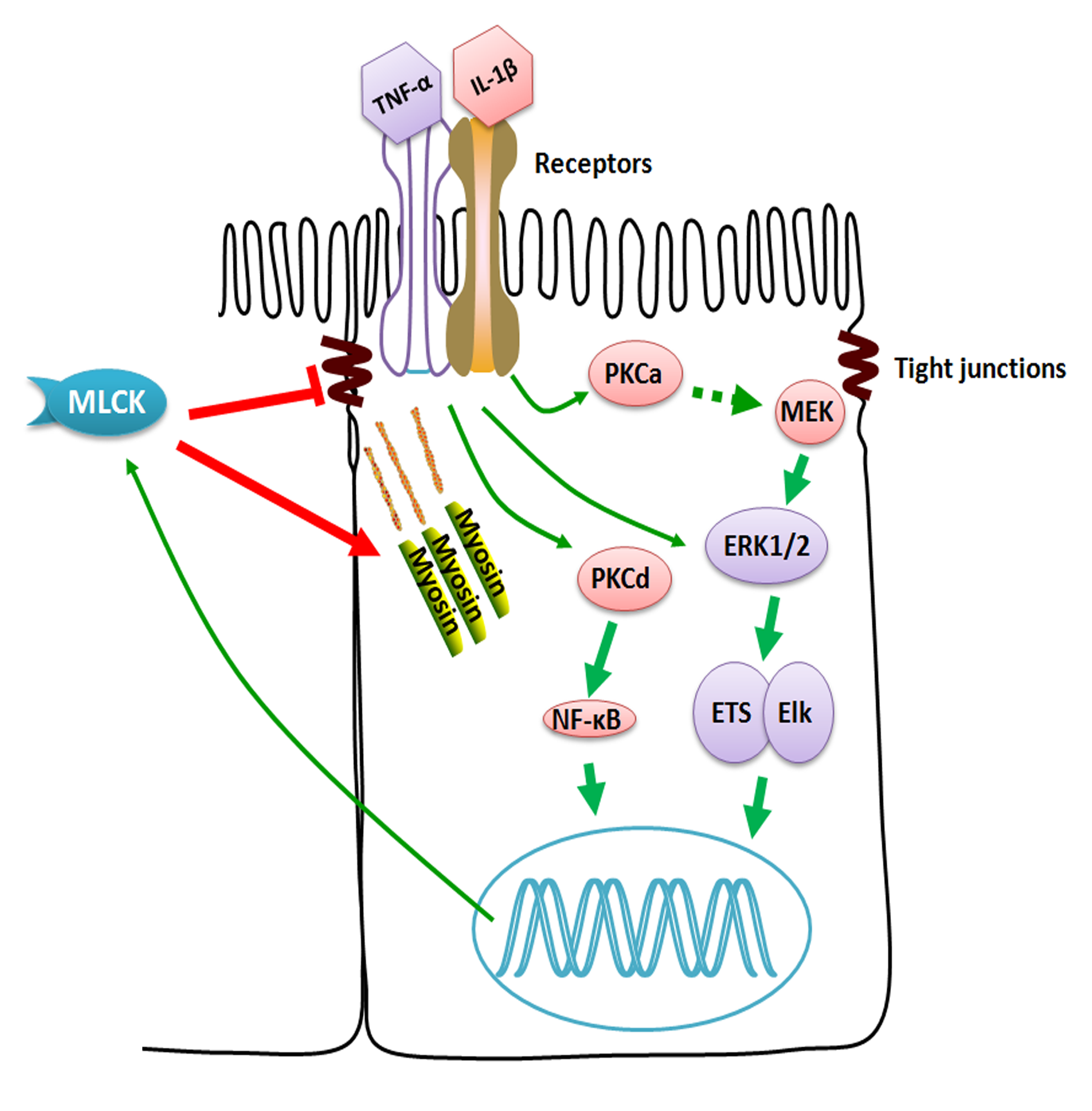

Supplement: FIGURE S1 — Mechanisms underlying MLCK-induced regulation of the endothelial barrier function are shown. Solid arrows indicate direct interaction and dotted arrows indicate indirect interactions. [file Image_1.tif]
